# C-reactive protein-albumin-lymphocyte (CALLY) index and all-cause and cardiovascular mortality in the general population: a national health and nutrition examination survey analysis

**DOI:** 10.1186/s13019-026-04479-x

**Published:** 2026-06-30

**Authors:** Xia-Ying Xu, Da-Hai Li, Yu-Cheng Li, Qing Ou, Hong-Yun Wei, Xiu-Ying Shi, Jing-Ya Xie, Da-Dong Yan, Zheng-Wei Zhao

**Affiliations:** 1 Department of Critical Care Medicine, Nanfang Hospital, Southern Medical University, Guangzhou, 510515 China; 2 Department of Dialysis Center, Nanfang Hospital, Southern Medical University, Guangzhou, 510515 China

**Keywords:** CALLY, C-reactive protein-albumin-lymphocyte, All-cause mortality, Cardiovascular mortality, Competing risk, Incremental predictive value

## Abstract

**Background:**

The C-reactive protein-albumin-lymphocyte (CALLY) index is a composite biomarker integrating systemic inflammation (elevated C-reactive protein), nutritional status (decreased albumin), and immune function (reduced lymphocyte count). Its prognostic significance for mortality in the general population remains insufficiently characterized.

**Methods:**

We analyzed data from the National Health and Nutrition Examination Survey (NHANES, 1999–2010), comprising 11,797 adults (5,671 men [weighted 48.4%] and 6,126 women [weighted 51.6%]; median age, 45 years). The CALLY index was calculated for each participant. Associations between the CALLY index and all-cause and cardiovascular mortality were evaluated using Kaplan-Meier analysis (for descriptive visualization), weighted multivariable Cox proportional hazards regression, Fine-Gray competing risk models, and restricted cubic spline (RCS) regression. Predictive performance was assessed using time-dependent receiver operating characteristic (ROC) analysis. The incremental predictive value beyond established risk factors was quantified using the integrated discrimination improvement (IDI) and category-free net reclassification improvement (NRI).

**Results:**

Compared with the lowest quartile, the highest CALLY quartile was associated with lower risks of all-cause mortality (multivariable-adjusted hazard ratio [HR], 0.61; 95% confidence interval [CI], 0.51–0.74; *P* < 0.001) and cardiovascular mortality (HR, 0.51; 95% CI, 0.39–0.68; *P* < 0.001). In Fine-Gray competing risk models, the subdistribution hazard ratio for cardiovascular mortality was 0.55 (95% CI, 0.39–0.76; *P* < 0.001), with a lower cumulative incidence of cardiovascular death in quartile 4 versus quartile 1 (Gray’s test, *P* < 0.001). RCS analyses revealed significant non-linear associations: an approximately L-shaped relationship for all-cause mortality and a monotonically decreasing non-linear pattern for cardiovascular mortality (both *P* for non-linearity < 0.001). The CALLY index provided modest incremental predictive value beyond established risk factors for all-cause mortality (IDI, 0.4%; *P* = 0.007), but individual-level risk reclassification was limited (continuous NRI, 2.7%; *P* = 0.272). Significant effect modification for all-cause mortality was observed only for alcohol use (*P* for interaction = 0.005).

**Conclusion:**

Higher CALLY index values were significantly associated with lower mortality risk. The CALLY index demonstrated a significant, independent, and non-linear inverse association with all-cause and cardiovascular mortality in US adults. Its standalone predictive discrimination was modest (time-dependent AUC < 0.70), but its integration into multivariable risk models provided modest yet statistically significant incremental prognostic information (IDI, 0.4%; *P* = 0.007). Individual-level risk reclassification was limited (continuous NRI, 2.7%; *P* = 0.272). These findings suggest that CALLY may serve as a complementary biomarker for risk stratification when integrated with conventional risk factors rather than as a standalone prediction tool.

**Supplementary Information:**

The online version contains supplementary material available at https://doi.org/10.1186/s13019-026-04479-x.

## Introduction

Cardiovascular disease (CVD) remains the leading cause of disability and mortality worldwide, accounting for approximately 17.9 million deaths annually—31% of all global fatalities [1]. Despite advances in diagnostic and therapeutic strategies, the burden of CVD-related mortality continues to rise, posing a substantial public health challenge [1, 2]. Accurate risk stratification, particularly among high-risk groups such as older adults and individuals with comorbidities, is essential for implementing effective early interventions and improving clinical outcomes. All-cause mortality serves as a fundamental and objective endpoint, providing a critical metric for evaluating population health status and intervention effectiveness.

Risk assessment tools commonly used in clinical practice, such as the Framingham Risk Score, primarily incorporate traditional risk factors, including age, blood pressure, lipid levels, and smoking history [3, 4]. However, these models have largely overlooked biomarkers reflecting the pathophysiological axes of CVD, particularly chronic inflammation, nutritional status, and immune dysregulation [5]. Accumulating evidence indicates that these three factors are interconnected and collectively promote atherosclerosis. C-reactive protein (CRP) is an established systemic inflammatory marker that directly contributes to endothelial damage [6]; serum albumin serves as a vital indicator of nutritional status and hepatic synthetic function, with low levels associated with poor prognosis [7]; and peripheral blood lymphocyte count is a straightforward parameter reflecting immune homeostasis and immunosurveillance [8].

Although existing composite biomarkers such as the prognostic nutritional index (PNI), CRP-to-albumin ratio (CAR), and systemic immune-inflammation index (SII) have demonstrated prognostic utility in various clinical settings, they primarily capture only one or two pathophysiological dimensions (e.g., inflammation-nutrition or inflammation-immunity). The CALLY index uniquely integrates all three axes—inflammation, nutrition, and immunity—into a single metric derived from routinely available laboratory parameters, potentially providing a more comprehensive risk assessment. Recent studies have begun evaluating CALLY in specific populations using the NHANES dataset. Zhang et al. (2026) examined the association between CALLY and mortality in adults with depression (*n* = 4,426) using conventional Cox proportional hazards regression and restricted cubic splines [9]; Han et al. (2025) investigated CALLY’s prognostic value in patients with prevalent CVD (*n* = 2,183) using similar conventional Cox models [10]; and Wu et al. (2025) analyzed the relationship between CALLY and peripheral artery disease in the general population using logistic and Cox regression [11]. However, these studies share several important methodological limitations. First, all employed conventional Cox regression without accounting for competing risks of non-cardiovascular death, which may overestimate cardiovascular mortality associations when competing events are common. Second, none formally quantified incremental predictive value using metrics such as IDI or NRI. Third, none conducted systematic time-dependent ROC analysis with optimism correction. Fourth, sensitivity analyses were limited in scope, with no evaluation of collider bias from disease variable adjustment, temporal stability across survey cycles, or independence from metabolic and oxidative stress pathways. Consequently, whether the CALLY index provides independent prognostic information in the general adult population, and the precise nature of its dose-response relationship with mortality, remain incompletely characterized.

The CALLY index is a composite metric that integrates inflammation (CRP), nutrition (albumin), and immune function (lymphocyte count) into a single value, calculated as albumin (g/L) × lymphocytes (10⁹/L) / [CRP (mg/L) × 10] [12]. This index is proposed to sensitively reflect an individual’s overall inflammation-nutrition-immunity status. In populations with chronic kidney disease, the CALLY index exhibits a significant inverse association with all-cause and CVD mortality, with accelerated biological aging as a mediating factor [13]. In patients with cancer, particularly esophageal cancer, the CALLY index has demonstrated strong predictive value for long-term survival [14]. In acute cardiovascular diseases, such as ST-elevation myocardial infarction and acute coronary syndrome, it has been associated with superior prognostic performance for mortality prediction [15, 16]. Furthermore, cross-sectional studies have reported associations between elevated CALLY index values and reduced risks of peripheral arterial disease and stroke [11, 17]. Despite these encouraging findings in specific patient groups, current research has primarily focused on pathological populations such as individuals with chronic kidney disease, cancer, or acute-phase cardiovascular conditions [18–20]. Notably, systematic validation through large-scale prospective cohort studies involving the general adult population—a key target for CVD prevention—is lacking.

Whether the CALLY index offers independent prognostic information beyond traditional risk factors in a general population context remains uncertain. Furthermore, the shape of its dose-response relationship with mortality—whether linear or non-linear and whether specific risk thresholds exist—has yet to be elucidated in large-scale epidemiological cohorts. To address these gaps, we analyzed data from six consecutive cycles of NHANES (1999–2010), linked with mortality follow-up through 2019.

In contrast to prior NHANES-based CALLY studies that relied on conventional Cox models without competing risk adjustment or incremental utility assessment, the present study implements a unified methodological framework that, to our knowledge, has not been simultaneously applied in this field. The primary objectives were: (1) to evaluate the association between the CALLY index and all-cause and cardiovascular mortality in a representative sample of US adults using weighted multivariable Cox proportional hazards regression and Fine-Gray subdistribution hazards models to account for competing risks; (2) to quantify incremental predictive value beyond established risk factors using IDI and NRI; (3) to characterize the dose-response relationship using restricted cubic splines with cautious interpretation of extreme values; and (4) to validate findings through comprehensive sensitivity analyses addressing reverse causation, temporal stability, metabolic confounding, and collider bias. By investigating these relationships within a population-based cohort with diverse baseline characteristics, we sought to enhance the generalizability of previous findings from specific disease populations and to provide rigorous evidence regarding the potential utility of the CALLY index as a complementary marker for mortality risk stratification.

## Methods

### Data source

The National Center for Health Statistics administers the NHANES database, which enrolled participants from 1999 to 2010. This biennially updated dataset includes questionnaire data and clinical and laboratory evaluations. Further information is available at www.cdc.gov/nchs/nhanes/index.htm. The Research Ethics Review Committee of the National Center for Health Statistics oversaw the study, ensuring that informed consent was obtained from all participants.

### Participant selection

**Fig. 1:**
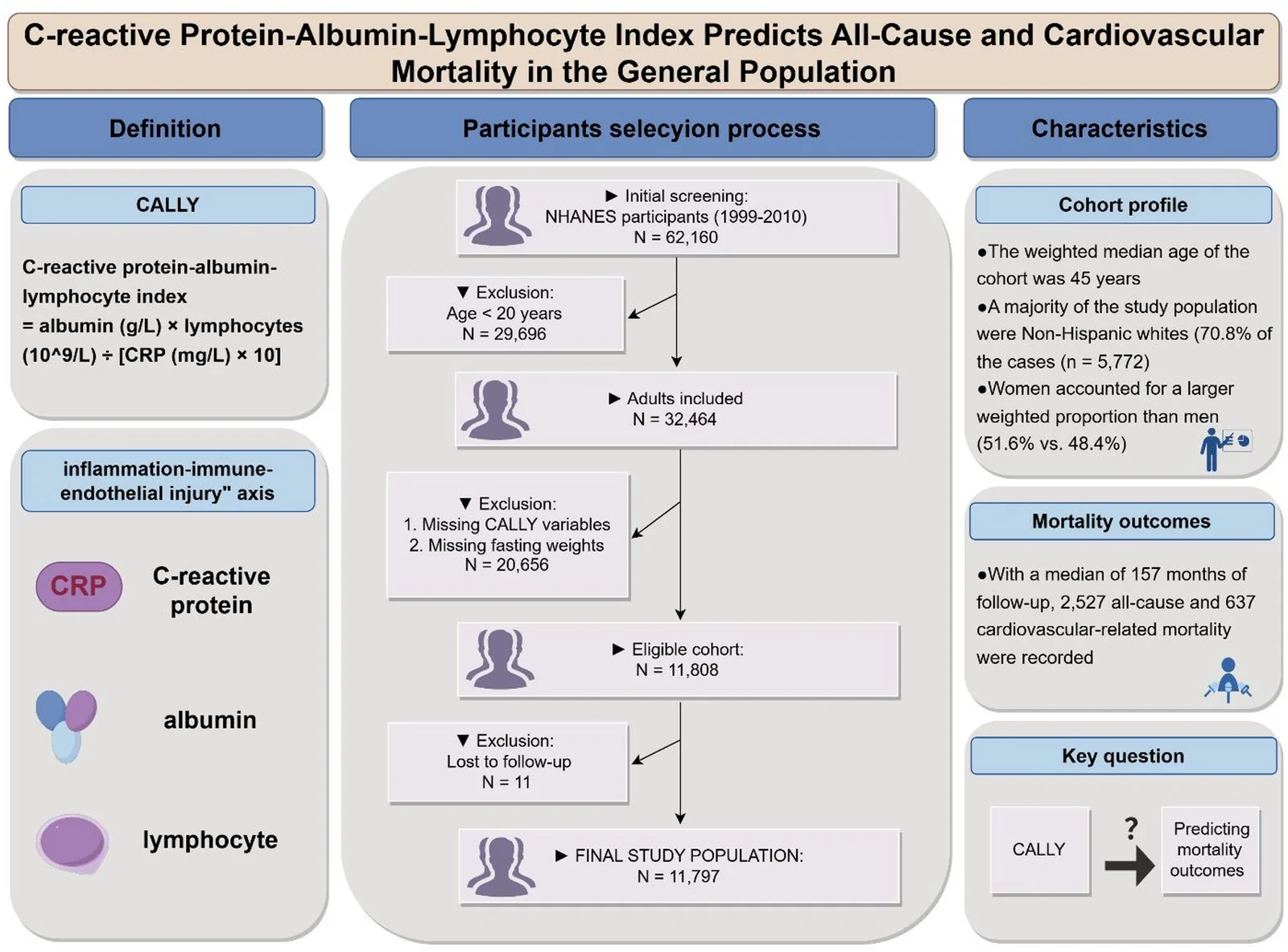
Flowchart of participant selection from NHANES 1999–2010. CALLY: C-reactive Protein-Albumin-Lymphocyte Index

The analysis sample initially comprised 62,160 participants from six biennial NHANES surveys conducted between 1999 and 2010. Participants aged < 20 years were first excluded (*n* = 29,696), leaving 32,464 adults. Among these, participants with missing exposure (lymphocyte count, albumin, or CRP), missing outcome (mortality) data, or missing anthropometric measurements were further excluded (*n* = 20,656), yielding 11,808 participants with complete exposure and outcome data. An additional 11 participants were lost to follow-up, resulting in a final analytic sample of 11,797 eligible adults (Fig. 1). Missing covariate values among the remaining participants (education [1.2%], smoking [2.8%], alcohol [3.1%], poverty income ratio [7.3%]) were imputed using multiple imputation by chained equations (MICE, 20 imputed datasets).

### Assessment of the CALLY index

Blood samples were collected at mobile examination centers and transported to centralized NHANES laboratories for analysis. Complete blood counts, including lymphocyte differentials, were performed using automated hematology analyzers according to standardized NHANES laboratory protocols. Serum albumin was measured by the bromocresol purple method, and serum CRP was quantified using latex-enhanced nephelometric immunoassay. The CALLY index was calculated as albumin (g/L) × lymphocytes (10⁹/L) / [CRP (mg/L) × 10]. To ensure nationally representative results, all statistical analyses incorporated survey weights to account for the complex multistage sampling design of NHANES. Based on the overall distribution of CALLY index values in the study population, participants were categorized into four quartiles (Q1: ≤1.54; Q2: >1.54–3.71; Q3: >3.71–9.40; Q4: ≥ 9.40), with Q1 serving as the reference group. Sex-nonspecific cutoffs were used to maintain consistency with previous literature and facilitate clinical interpretability.

### Assessment of mortality outcomes

The follow-up period was defined as the interval between the baseline interview and the date of death, ascertained through linkage with the National Death Index (https://www.cdc.gov/nchs/data-linkage/mortality-public). Mortality follow-up extended through December 31, 2019. Underlying causes of death were classified according to the *International Classification of Diseases*,* Tenth Revision* (ICD-10), following US National Center for Health Statistics protocols.

### Assessment of covariates

Demographic and clinical characteristics were extracted from the NHANES database. Distributions of continuous variables are presented in Supplementary Figure S1. Socioeconomic variables included race/ethnicity (Mexican American, other Hispanic, non-Hispanic White, non-Hispanic Black, or other), sex, age, educational attainment (high school or less, some college, or college graduate or above), marital status (single, married or cohabiting, or widowed/divorced/separated), and poverty income ratio (< 1.3, 1.3–3.5, or > 3.5). Health insurance status was also recorded. Lifestyle factors comprised smoking status (never: <100 cigarettes in a lifetime; former: ≥100 cigarettes but quit; current: ≥100 cigarettes or smoking daily) and alcohol consumption (abstinence: no alcohol in the past year; consumption: any alcohol intake in the preceding 12 months). Comorbidities were defined as follows. Hypertension was diagnosed based on blood pressure measurements (systolic ≥ 140 mmHg or diastolic ≥ 90 mmHg) or physician diagnosis according to the Seventh Report of the Joint National Committee criteria. Diabetes mellitus was defined by any of the following: fasting plasma glucose ≥ 7.0 mmol/L, glycated hemoglobin ≥ 6.5%, self-reported use of antidiabetic medication or insulin, or physician diagnosis per American Diabetes Association criteria. CVD and malignant tumors were recorded as binary variables (yes/no). Body mass index (BMI) was calculated as weight in kilograms divided by height in meters squared and categorized as underweight (≤ 18.5 kg/m²), normal weight (18.5–24.9 kg/m²), overweight (25–29.9 kg/m²), or obese (≥ 30 kg/m²). Follow-up duration was calculated from the baseline examination until death or censoring.

To guide covariate adjustment, we constructed a directed acyclic graph (DAG) based on prior clinical knowledge and published literature (Supplementary Figure S8). In this DAG, the exposure was the CALLY index and the outcome was all-cause mortality. The following variables were identified as common causes of both exposure and outcome (confounders): age, sex, BMI, smoking, alcohol use, baseline CVD, cancer, diabetes, hypertension, race/ethnicity, educational attainment, health insurance status, and marital status. These confounders were included in multivariable Cox regression models to estimate the total effect of the CALLY index on mortality. We employed a three-tiered hierarchical modeling approach for both all-cause and cardiovascular mortality. Model 1 was unadjusted. Model 2 was adjusted for age, sex, and race/ethnicity. Model 3 was further adjusted for marital status, educational attainment, BMI, poverty income ratio, smoking status, alcohol consumption, CVD, hypertension, cancer, diabetes, and health insurance status. Prior to model fitting, multicollinearity was assessed using variance inflation factors; all values were well below the conventional threshold of 5 (Supplementary Table S1). Sensitivity analyses excluding highly correlated covariates yielded consistent results. The following variables were not included owing to unavailability or insufficient granularity in the NHANES 1999–2010 cycles: detailed physical activity metrics, comprehensive dietary intake data, and medication use (e.g., statins, anti-inflammatory agents).

### Statistical analysis

To ensure accurate variance estimation and national representativeness of the US population, this study incorporated sample weights and stratification into all analyses. Participants were categorized into four CALLY quartiles: Q1 (CALLY ≤ 1.54), Q2 (1.54 < CALLY ≤ 3.71), Q3 (3.71 < CALLY ≤ 9.40), and Q4 (CALLY ≥ 9.40). Continuous variables are reported as medians with interquartile ranges, and categorical variables as frequencies and weighted percentages (Table 1). Cox proportional hazards models were used to examine associations between the CALLY index and mortality outcomes, with detailed model specifications provided in Sect.  2.5.

**Table 1:** The demographic characteristics of adults in the present study

Variables	Total (*n* = 11,797)	Survivors (*N* = 9,270, 84.9%)	Non-survivors (*N* = 2,527, 15.1%)	*P*-value
**Age**,** M (Q1**,** Q3)**	45.00 (32.00–58.00)	42.00 (31.00–53.00)	69.00 (56.00–78.00)	< 0.001
**Follow-up time**,** M (Q1**,** Q3)**	157.00 (127.00–214.00)	164.00 (135.00–219.00)	99.00 (58.00–144.00)	< 0.001
**BMI**,** M (Q1**,** Q3)**	27.25 (23.80–31.60)	27.23 (23.78–31.53)	27.35 (23.96–31.84)	0.235
**Albumin (g/L)**,** M (Q1**,** Q3)**	43.00 (41.00–45.00)	43.00 (41.00–45.00)	42.00 (40.00–44.00)	< 0.001
**CRP (mg/dL)**,** M (Q1**,** Q3)**	0.19 (0.08–0.44)	0.18 (0.07–0.41)	0.28 (0.13–0.66)	< 0.001
**Lymphocytes (1000 cell/µL)**,** M (Q1**,** Q3)**	1.90 (1.60–2.30)	1.90 (1.60–2.30)	1.80 (1.40–2.30)	< 0.001
**CALLY index**,** M (Q1**,** Q3)**	4.29 (1.78–10.81)	4.74 (1.96–11.81)	2.73 (1.09–5.85)	< 0.001
**Gender**,** n (%)**				0.006
Male	5,671 (48.4)	4,276 (47.7)	1,395 (52.0)	
Female	6,126 (51.6)	4,994 (52.3)	1,132 (48.0)	
**Age**,** n (%)**				< 0.001
20–34	3,082 (29.1)	3,012 (33.4)	70 (4.7)	
35–48	2,819 (29.5)	2,660 (32.9)	159 (10.7)	
49–64	2,958 (25.3)	2,416 (25.3)	542 (25.4)	
>64	2,938 (16.1)	1,182 (8.4)	1,756 (59.3)	
**Race**,** n (%)**				< 0.001
Mexican American	2,465 (7.8)	2,114 (8.5)	351 (3.6)	
Other Hispanic	900 (5.4)	793 (5.7)	107 (3.6)	
Non-Hispanic White	5,772 (70.8)	4,207 (69.2)	1,565 (79.6)	
Non-Hispanic Black	2,199 (10.7)	1,754 (10.8)	445 (9.9)	
Other race	461 (5.4)	402 (5.7)	59 (3.3)	
**BMI**,** n (%)**				0.245
<18.5	183 (1.8)	130 (1.8)	53 (1.9)	
18.5–24.9	3,371 (31.6)	2,644 (31.9)	727 (29.7)	
25-29.9	4,133 (33.3)	3,246 (33.3)	887 (33.0)	
≥ 30	4,110 (33.4)	3,250 (33.0)	860 (35.4)	
**Education level**,** n (%)**				< 0.001
≤High school	6,402 (44.6)	4,743 (41.9)	1,659 (59.3)	
College	3,139 (29.9)	2,597 (30.6)	542 (26.0)	
> College	2,256 (25.5)	1,930 (27.4)	326 (14.6)	
**Marital status**,** n (%)**				< 0.001
Married/living with partner	7,279 (65.1)	5,925 (66.4)	1,354 (57.5)	
Widowed/divorced/separated	2,724 (18.8)	1,712 (15.7)	1,012 (36.0)	
Never married	1,794 (16.1)	1,633 (17.8)	161 (6.5)	
**PIR**,** n (%)**				< 0.001
<1.3	3,762 (22.2)	2,834 (21.0)	928 (29.1)	
1.3–3.5	4,408 (36.2)	3,342 (34.9)	1,066 (43.8)	
>3.5	3,627 (41.6)	3,094 (44.2)	533 (27.1)	
**CALLY_Q**,** n (%)**				< 0.001
Q1 (≤ 1.54)	2,951 (21.5)	2,075 (19.3)	876 (33.9)	
Q2 (1.54–3.71)	2,948 (23.8)	2,238 (23.1)	710 (27.2)	
Q3 (3.71–9.40)	2,961 (26.1)	2,390 (26.5)	571 (23.9)	
Q4 (≥ 9.40)	2,937 (28.6)	2,567 (31.0)	370 (15.0)	
**Smoking**,** n (%)**				< 0.001
Never	6,186 (51.9)	5,154 (54.2)	1,032 (38.9)	
Ever	3,070 (25.5)	2,109 (23.4)	961 (36.9)	
Now	2,541 (22.7)	2,007 (22.4)	534 (24.2)	
**Insurance status**,** n (%)**	9,089 (81.2)	6,818 (79.6)	2,271 (90.4)	< 0.001
**Alcohol use**,** n (%)**	9,072 (81.0)	7,465 (83.7)	1,607 (66.2)	< 0.001
**Cancer**,** n (%)**	1,055 (8.4)	549 (6.3)	506 (20.6)	< 0.001
**CVD**,** n (%)**	1,031 (6.7)	422 (3.7)	609 (23.1)	< 0.001
**Hypertension**,** n (%)**	2,327 (15.0)	1,319 (11.4)	1,008 (34.8)	< 0.001
**Diabetes**,** n (%)**	1,853 (10.9)	1,084 (8.2)	769 (26.0)	< 0.001

M, median; Q, quartile; CALLY, C-reactive protein-albumin-lymphocyte index; CRP, C-reaction protein; BMI, body mass index; PIR, poverty income ratio; CVD, cardiovascular disease; Non-normally distributed variables are displayed as median with 1st and 3rd quartile (M, Q1, Q3); Categorical variables are displayed as numbers with weighted percentages (n, %). Bold values indicate statistical significance (p<0.05)

Missing data handling is detailed in Sect.  2.2. Briefly, participants with incomplete exposure or outcome data were excluded, and missing covariate data among the remaining participants were imputed using MICE (20 imputed datasets).

Survival analyses used Kaplan-Meier curves with log-rank tests to compare survival patterns across CALLY quartiles. These curves represent unadjusted descriptive visualizations and should not be interpreted as measures of independent predictive discrimination. Predictive performance was assessed using time-dependent ROC analysis with inverse probability of censoring weighting (IPCW). The optimism-corrected C-index was calculated based on 500 bootstrap resamples.

#### Competing risk analysis

For cardiovascular mortality, we applied the Fine-Gray subdistribution hazards model [21] to account for the competing risk of death from non-cardiovascular causes. This approach estimates the subdistribution hazard ratio (SHR) while treating non-cardiovascular death as a competing event, providing valid inference for the cumulative incidence of the event of interest [21, 22]. Cumulative incidence functions (CIFs) were compared across CALLY quartiles using Gray’s test.

#### Incremental predictive value assessment

To evaluate whether the CALLY index improved risk prediction beyond established risk factors (Model 3 covariates), we calculated the IDI and NRI. The base model included age, sex, race/ethnicity, BMI, education, marital status, poverty income ratio, smoking, alcohol, hypertension, diabetes, CVD, cancer, and insurance. The extended model added the CALLY index (continuous) to the base model. The IDI was computed as the difference in discrimination slopes between models. We report continuous NRI (cNRI) and category-free NRI. Bootstrapping (500 resamples) was used to derive 95% confidence intervals and *P* values.

#### Dose-response analysis

Dose-response analysis was conducted using RCS regression for all-cause and cardiovascular mortality. For each outcome, five knots were positioned at the 5th, 27.5th, 50th, 72.5th, and 95th percentiles of the CALLY distribution. The specific knot locations varied slightly between models: for all-cause mortality, knots were placed at 0.44, 1.71, 3.71, 8.36, and 34.4; for cardiovascular mortality, at 0.48, 1.82, 3.98, 9.01, and 36.9. The reference value was set at the 50th percentile (CALLY ≈ 3.8) for both models. Software-generated nadirs and their confidence intervals are reported, although their interpretation warrants caution owing to data sparsity beyond the upper knot positions.

#### Subgroup and interaction analyses

Subgroup and interaction analyses were conducted across demographic and health-related categories, including age groups (20–34, 35–48, 49–64, and > 64 years), sex, educational attainment, marital status, race/ethnicity, poverty income ratio, BMI (18.5–24.9, 25–29.9, and ≥ 30 kg/m²), smoking status, alcohol use, and medical history (hypertension, cancer, diabetes, and CVD). The likelihood ratio test was used to evaluate interactions across subgroups.

#### Sensitivity analyses

To confirm the robustness of the primary findings, four sensitivity analyses were conducted. First, to address potential reverse causality, participants who died within two years of baseline were excluded. Second, to evaluate temporal stability, the analysis was restricted to participants from 1999 to 2002. Third, to assess independence from metabolic pathways, additional adjustments were made for total bilirubin and serum uric acid. Fourth, to evaluate potential collider bias from adjusting for prevalent diseases, we fitted Cox models excluding CVD, cancer, hypertension, and diabetes sequentially. Participants with missing biomarker data were excluded from this sensitivity analysis. All statistical analyses were performed using DecisionLinnc software (version 1.1.7.4).

## Results

### Baseline characteristics of adult participants

From 1999 to 2010, a total of 11,797 individuals were included in the study. The median age was 45 years, and the median BMI was 27.25 kg/m². The study population comprised 5,671 (48.4%) men and 6,126 (51.6%) women. The majority of participants were non-Hispanic White (70.8%, *n* = 5,772), whereas non-Hispanic Black participants accounted for 10.7% (*n* = 2,199). More than 50% of participants had educational attainment beyond high school. In addition, 22.7% were current smokers, and 81.0% reported alcohol use. Regarding comorbidities, 10.9% reported a history of diabetes mellitus, 6.7% had CVD, and 8.4% had cancer. The insurance coverage rate was 81.2%, and the median CALLY index was 4.29. Over a median follow-up of 157 months, 2,527 deaths from various causes occurred, of which 637 were attributed to CVD. Compared with survivors, non-survivors were predominantly older men, primarily non-Hispanic White, with lower educational attainment. They were more likely to be widowed, divorced, or separated; had pre-existing health conditions; exhibited higher smoking rates; had lower socioeconomic status; and had lower CALLY index values (*P* < 0.01 for all comparisons; Table 1).

### Associations between the CALLY index and mortality in adults

**Fig. 2:**
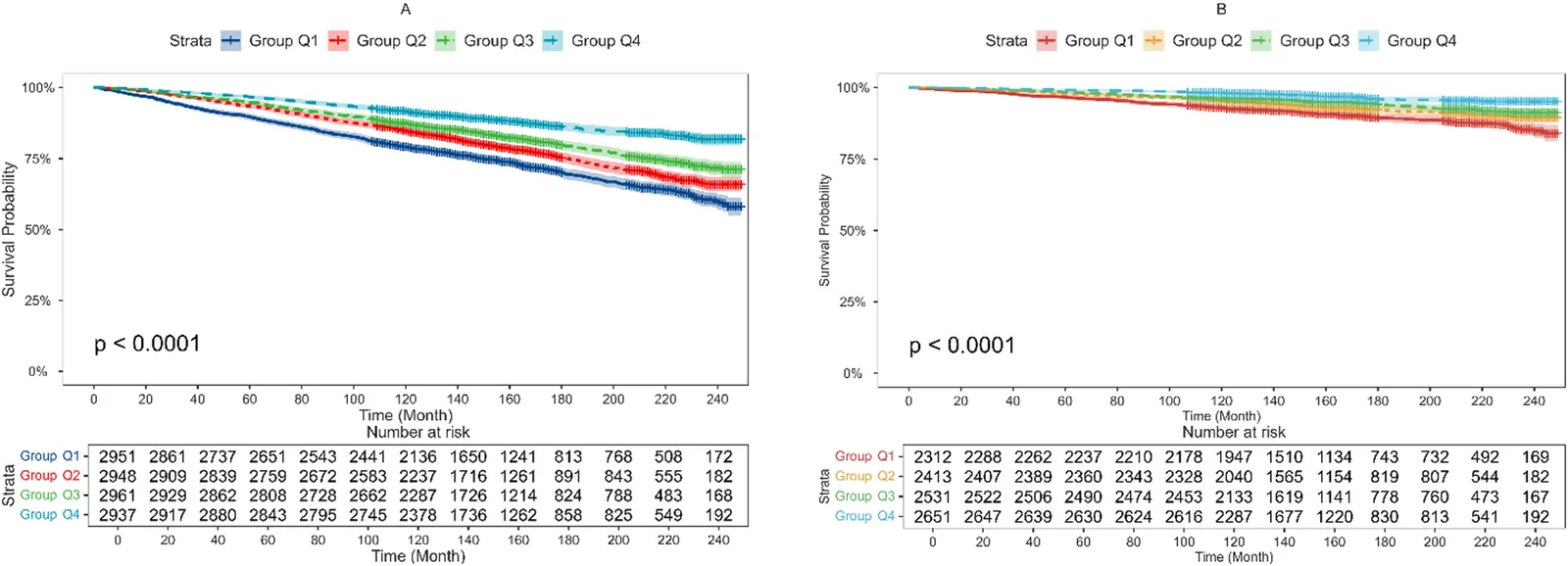
Kaplan-Meier survival curves by CALLY quartiles. (**A**) All-cause mortality; (**B**) Cardiovascular mortality. P values from log-rank tests are shown. These curves represent unadjusted, descriptive visualizations of survival patterns and should not be interpreted as measures of independent predictive discrimination. Number at risk reflects participants remaining in the study at each time point; for cardiovascular mortality (panel B), this includes only those alive and free of competing events

During follow-up (median 157 months, maximum 240 months), Kaplan-Meier analyses revealed significant differences across CALLY quartiles for both all-cause mortality (Fig. 2A) and cardiovascular mortality (Fig. 2B; both log-rank *P* < 0.0001). For all-cause mortality, pronounced survival stratification emerged over time, with 240-month survival rates of approximately 58% in Q1 versus 82% in Q4, corresponding to mortality rates of approximately 42% versus 18%. Cardiovascular mortality exhibited more gradual separation between groups, with consistently higher overall survival probabilities throughout follow-up, reflected in 240-month survival rates of approximately 88% in Q1 and 95% in Q4.

**Fig. 3:**
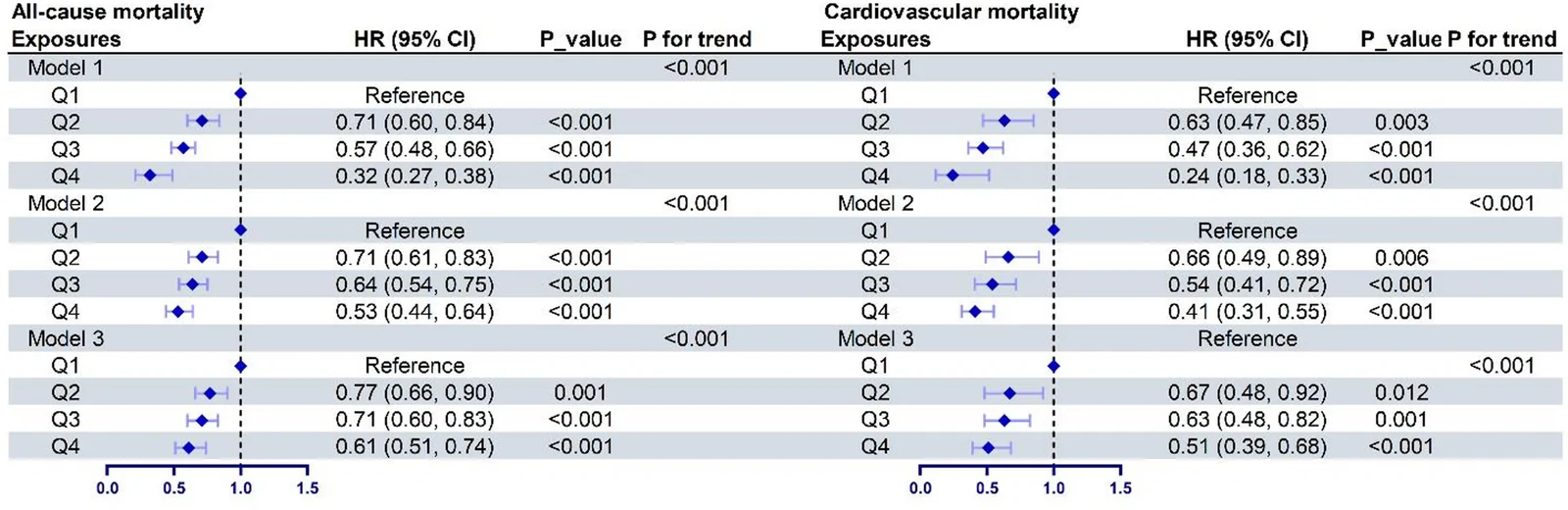
Forest plots reveal the connection between CALLY indices and the mortality rates from all causes and cardiovascular diseases in adults. CALLY: C-reactive Protein-Albumin-Lymphocyte Index; Q, quartile. Model 1: unadjusted; Model 2: adjusted for age, sex, and race/ethnicity; Model 3: adjusted for age, sex, race/ethnicity, marital status, educational attainment, BMI, PIR, smoking status, alcohol consumption, cardiovascular disease, hypertension, cancer, diabetes, and insurance coverage

In weighted Cox proportional hazards regression, higher CALLY quartiles demonstrated a consistent inverse relationship with mortality risk across all adjustment levels (Fig. 3). In Model 1 (unadjusted), the HR for all-cause mortality comparing Q4 with Q1 was 0.32 (95% CI, 0.27–0.38), and for cardiovascular mortality, 0.24 (95% CI, 0.18–0.33; both *P* < 0.001). After adjustment for age, sex, and race/ethnicity in Model 2, the HR for Q4 was 0.53 (95% CI, 0.44–0.64) for all-cause mortality and 0.41 (95% CI, 0.31–0.55) for cardiovascular mortality (both *P* < 0.001). In the fully adjusted Model 3, significant associations persisted, with HRs of 0.61 (95% CI, 0.51–0.74) for all-cause mortality and 0.51 (95% CI, 0.39–0.68) for cardiovascular mortality (both *P* < 0.001). The decrease in effect size from Model 1 to Model 3 indicates confounding by age and comorbidities, while the persistent association in fully adjusted models supports an association independent of the measured covariates.

#### Competing risk analysis for cardiovascular mortality

**Fig. 4:**
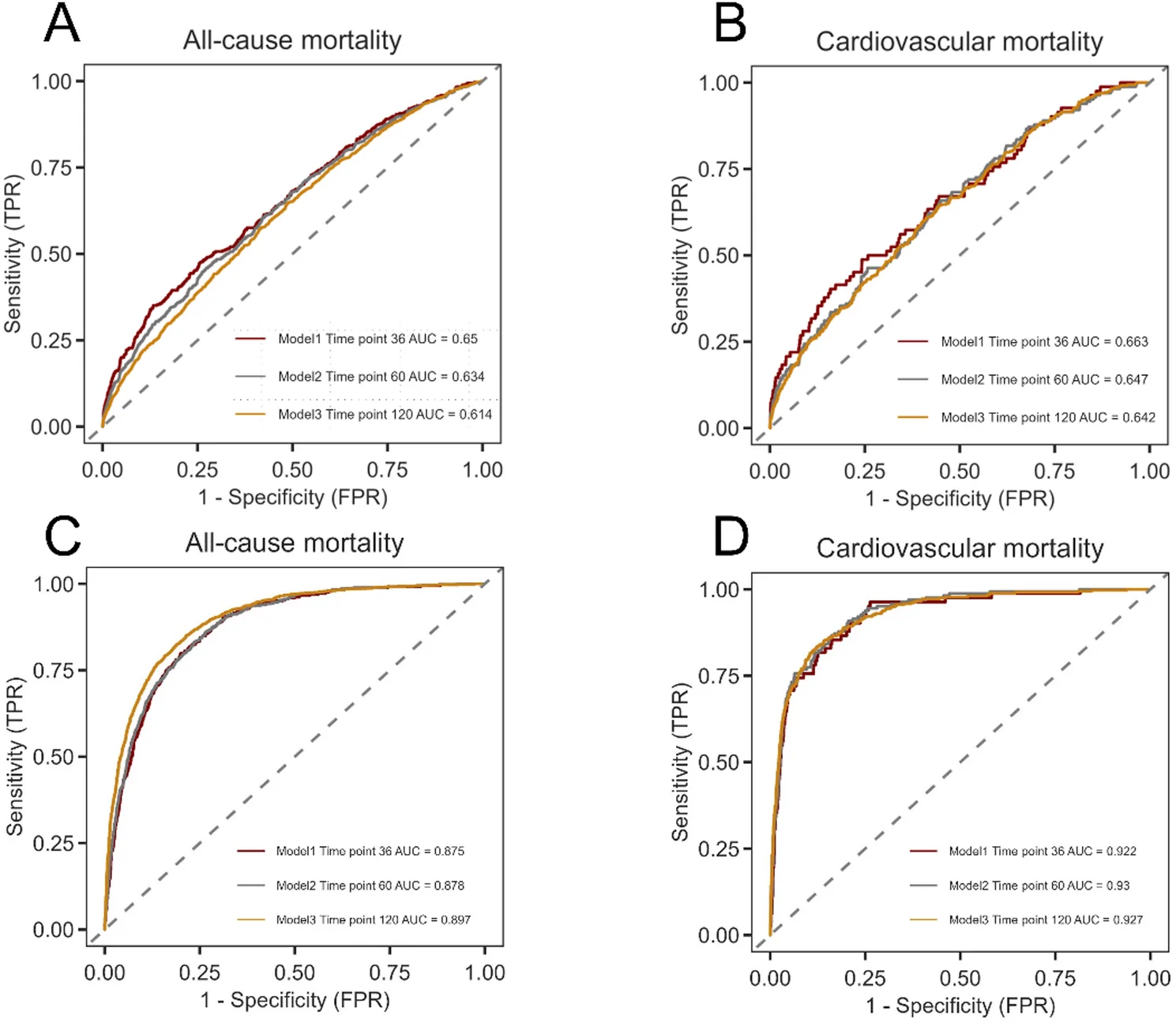
Time-dependent receiver operating characteristic (ROC) curves for the CALLY index in predicting mortality outcomes. (**A**,** B**) Univariable analysis of CALLY alone without covariate adjustment: (**A**) all-cause mortality; (**B**) cardiovascular mortality. (**C**,** D**) Multivariable analysis in the full cohort adjusted for age, sex, race/ethnicity, body mass index, educational attainment, poverty income ratio, marital status, smoking status, alcohol consumption, hypertension, diabetes mellitus, cardiovascular disease, cancer, and insurance coverage. Optimism-corrected area under the curve (AUC) values were derived from 500 bootstrap resamples. AUC values are reported for three time points (36, 60, and 120 months)

**Fig. 5:**
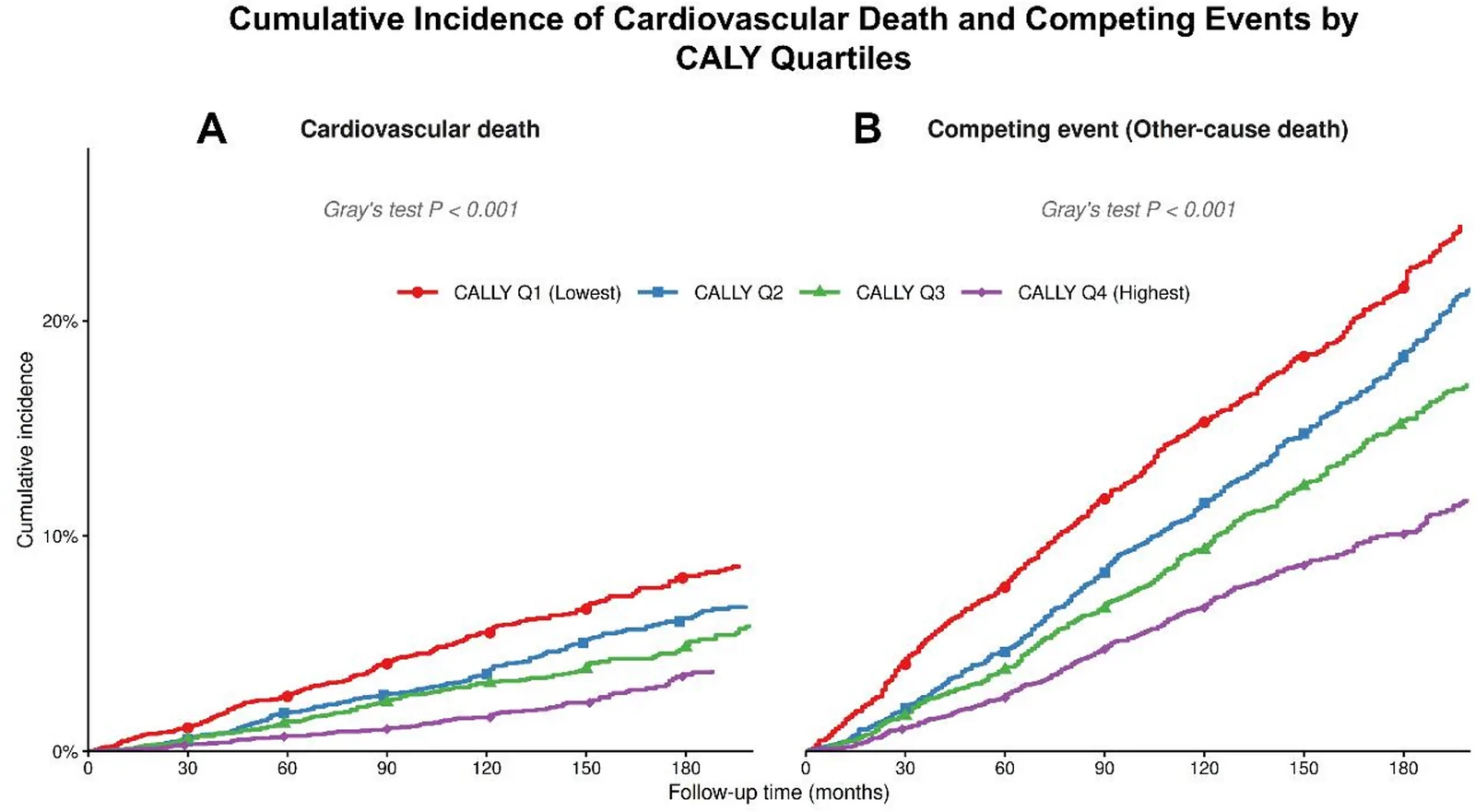
Cumulative incidence of cardiovascular death and competing events by CALLY quartiles. (**A**) Cumulative incidence of cardiovascular death; (**B**) Cumulative incidence of non-cardiovascular death (competing event). Gray’s test *P* < 0.001 for both panels. Cumulative incidence functions were estimated using the Aalen-Johansen method. Q1 (red): CALLY ≤ 1.54; Q2 (blue): 1.54 < CALLY ≤ 3.71; Q3 (green): 3.71 < CALLY ≤ 9.40; Q4 (purple): CALLY ≥ 9.40. At 180 months, cumulative incidence of cardiovascular death was 8.14% (Q1), 6.19% (Q2), 4.91% (Q3), and 3.56% (Q4); cumulative incidence of non-cardiovascular death was 21.78% (Q1), 18.57% (Q2), 15.36% (Q3), and 10.18% (Q4)

**Fig. 6:**
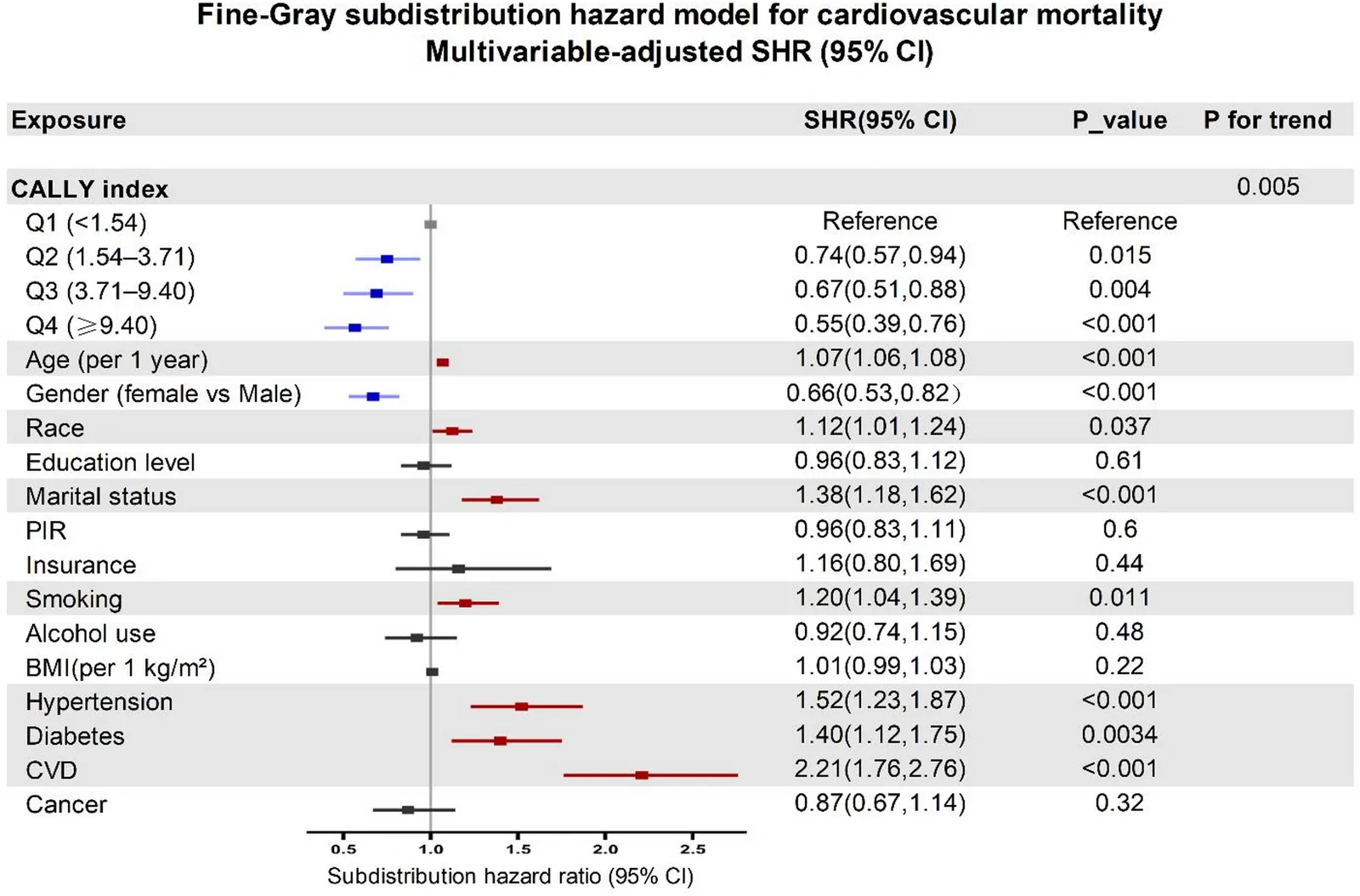
Fine-Gray subdistribution hazard model for cardiovascular mortality. Multivariable-adjusted subdistribution hazard ratios (SHR) and 95% confidence intervals are shown. The model was adjusted for age, sex, race/ethnicity, marital status, educational attainment, BMI, poverty income ratio, smoking status, alcohol consumption, hypertension, diabetes, CVD, cancer, and insurance coverage. Red squares indicate risk factors (*P* < 0.05); blue squares indicate **protective associations** (*P* < 0.05); gray squares indicate non-significant associations

After accounting for the competing risk of non-cardiovascular death using Fine-Gray subdistribution hazards models, the association between the CALLY index and cardiovascular mortality remained significant and directionally consistent with the standard Cox model. In the fully adjusted model, participants in Q2 had an SHR of 0.74 (95% CI, 0.57–0.94; *P* = 0.015), Fig. 4, those in Q3 had an SHR of 0.67 (95% CI, 0.51–0.88; *P* = 0.004), and those in Q4 had an SHR of 0.55 (95% CI, 0.39–0.76; *P* < 0.001) compared with Q1. The dose-response trend across quartiles persisted (*P* for trend = 0.005). By 180 months of follow-up, the cumulative incidence of cardiovascular death was 8.14% in Q1 versus 3.56% in Q4, whereas the cumulative incidence of non-cardiovascular death was 21.78% in Q1 versus 10.18% in Q4 (both Gray’s test, *P* < 0.001). Cumulative incidence functions are illustrated in Fig. 5, with corresponding event counts and percentages provided in Table 2. The multivariable-adjusted Fine-Gray SHRs are displayed in Fig. 6. The magnitude of association was comparable to that from the standard Cox model (HR, 0.51 for Q4), suggesting that the observed association is not materially biased by competing risks. Detailed cumulative incidence estimates by quartile and follow-up time point are provided in Supplementary Table S3.

**Table 2:** CALLY Quartile x Death Event Cross-tabulation

Group	Total *N*	Surviving (censored)	CVD death	Other-cause death	Total death
Q1 (Lowest)	2951	2075 (*70.32%*)	237 (8.03%)	639 (*21.65%*)	876 (*29.68%*)
Q2	2948	2238 (*75.92%*)	175 (*5.94%*)	535 (*18.15%*)	710 (*24.08%*)
Q3	2961	2390 (*80.72%*)	141 (*4.76%*)	430 (*14.52%*)	571 (*19.28%*)
Q4 (Highest)	2937	2567 (*87.40%*)	84 (*2.86%*)	286 (*9.74%*)	370 (*12.60%*)
**Total**	**11,797**	**9270** (***78.58%***)	**637 (5.40%)**	**1890 (16.02%)**	**2527** (***21.42%***)

M, median; Q, quartile; CALLY, C-reactive protein-albumin-lymphocyte index; CRP, C-reaction protein; BMI, body mass index; PIR, poverty income ratio; CVD, cardiovascular disease; Non-normally distributed variables are displayed as median with 1st and 3rd quartile (M, Q1, Q3); Categorical variables are displayed as numbers with weighted percentages (n, %). Bold values indicate statistical significance (p<0.05)

### Predictive performance and dose-response relationship of the CALLY index with mortality

Time-dependent ROC analysis with IPCW was conducted to assess discriminative ability at 3-, 5-, and 10-year intervals (36, 60, and 120 months). AUC values were calculated for both the full multivariable model (Model 3 covariates) and the CALLY index alone. The CALLY index alone exhibited limited discrimination, with AUC values below 0.70. For all-cause mortality, AUC values were 0.650 at 36 months, 0.634 at 60 months, and 0.614 at 120 months (Fig. 4A). For cardiovascular mortality, corresponding AUC values were 0.663 at 36 months, 0.647 at 60 months, and 0.642 at 120 months (Fig. 4B). After comprehensive adjustment for established risk factors (Model 3 covariates), discriminative ability improved substantially. In the full cohort (Fig. 4C, D), optimism-corrected AUC values derived from 500 bootstrap resamples were 0.875, 0.878, and 0.897 for all-cause mortality at 36, 60, and 120 months, respectively. For cardiovascular mortality, corresponding AUC values were 0.922, 0.930, and 0.927. Using a 70% random subsample of the cohort (Supplementary Figure S7), AUC values were 0.867, 0.874, and 0.898 for all-cause mortality and 0.936, 0.939, and 0.925 for cardiovascular mortality, consistent with the primary analysis and confirming model stability. Calibration assessment indicated adequate agreement between predicted and observed outcomes. The high AUC values (> 0.87) primarily reflect the substantial predictive capacity of established risk factors—specifically age, sex, and comorbidities—in the multivariable model, whereas the incremental contribution of the CALLY index alone was modest.

#### Incremental predictive value assessment

When added to the base model of established risk factors, the CALLY index provided a modest but statistically significant improvement in discrimination. For all-cause mortality, the IDI was 0.4% (95% CI: 0.1%–0.7%, *P* = 0.007), and for cardiovascular mortality, the IDI was 0.1% (95% CI: 0.0%–0.3%, *P* = 0.007). The identical *P* values reflect similar effect sizes and precision in bootstrap estimates for both outcomes within the same cohort (Supplementary Figure S9). However, NRI was not statistically significant for either outcome (all-cause: NRI = 2.7% [95% CI: − 2.2% to 7.6%], *P* = 0.272; cardiovascular: NRI = 1.3% [95% CI: − 1.7% to 4.3%], *P* = 0.399). The median improvement in predicted probabilities was significant for both outcomes (all-cause: 0.8% [95% CI: 0.4%–1.3%], *P* < 0.001; cardiovascular: 0.4% [95% CI: 0.1%–0.8%], *P* < 0.001), indicating that the CALLY index shifted the average predicted risk in the correct direction despite limited reclassification at the individual level. These incremental predictive value metrics are illustrated in Supplementary Figure S9. These results collectively confirm that the CALLY index contributes statistically significant but modest incremental discrimination beyond traditional risk factors, with limited individual-level reclassification utility.

#### Dose-response analysis

To examine the non-linear relationship between the CALLY index and mortality outcomes, RCS regression was conducted separately for each outcome, with five knots at the 5th, 27.5th, 50th, 72.5th, and 95th percentiles. Knot placements were: for all-cause mortality, 0.44, 1.71, 3.71, 8.36, 34.4; for cardiovascular mortality, 0.48, 1.82, 3.98, 9.01, 36.9. The reference was set at the 50th percentile (CALLY ≈ 3.8) for both models.

**Fig. 7:**
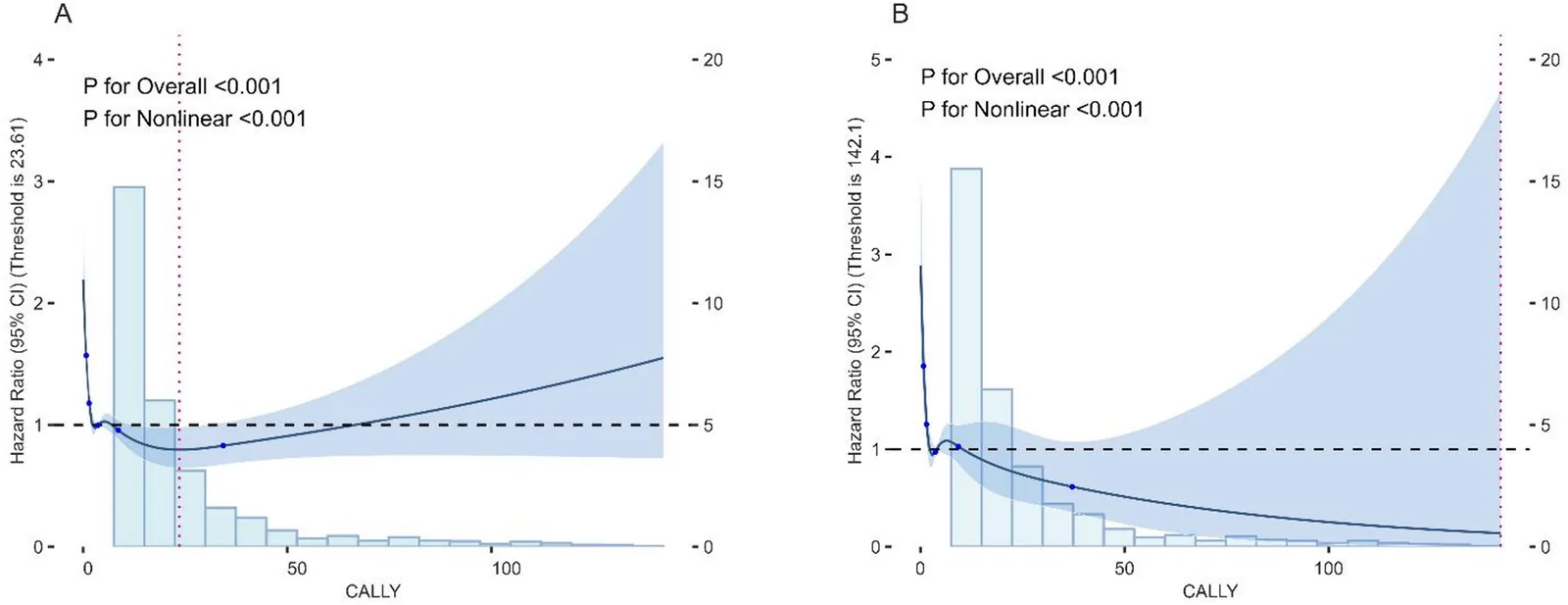
Restricted cubic spline analysis of the association between the CALLY index and mortality outcomes. (**A**) All-cause mortality; (**B**) Cardiovascular mortality. Adjusted for all covariates in Model 3. Five knots at the 5th, 27.5th, 50th, 72.5th, and 95th percentiles. Reference at the 50th percentile (CALLY ≈ 3.8). Histograms show CALLY distribution; shaded areas are 95% CIs. All-cause mortality shows a steep decline then plateau, with an unstable upturn at extreme values (CALLY > 50, *n* < 10). Cardiovascular mortality shows a monotonic non-linear inverse association. No reliable risk threshold or clinically interpretable nadir could be identified

For all-cause mortality (Fig. 7A), a significant non-linear association was identified (overall *P* < 0.001; *P* for non-linearity < 0.001). The fitted curve exhibited an approximately L-shaped pattern: risk declined steeply from low CALLY values and reached its lowest point in the moderate range (approximately CALLY 5–15, HR ≈ 0.8), after which it remained relatively flat across the bulk of the observed distribution. The software-estimated nadir was located at CALLY = 23.6; however, this estimate fell within the plateau phase rather than at the apparent minimum, and its 95% confidence interval was extremely wide and crossed the null (HR = 1), indicating statistical instability. Beyond the 95th percentile (CALLY > 34.4, *n* = 142 [1.2%]), the curve displayed an apparent upturn, but this was driven by fewer than 10 participants with CALLY > 50 and should be interpreted as a statistical artifact rather than a genuine biological reversal of risk.

For cardiovascular mortality (Fig. 7B), a significant non-linear association was also identified (overall *P* < 0.001; *P* for non-linearity < 0.001). The curve declined monotonically within the observed data range, with no evidence of a plateau or upturn. The software-estimated nadir at CALLY = 142.1 fell far outside the distribution of the data (only 8 participants [0.07%] had CALLY > 100) and is therefore clinically uninterpretable.

For both outcomes, no reliable risk threshold could be derived. The inverse associations were approximately L-shaped, with substantial risk reduction in the moderate CALLY range and no evidence of harm within the reliable data range. These findings corroborate the quartile-based results but do not support the identification of specific clinical cut-points.

### Subgroup and sensitivity analyses

We examined the consistency of CALLY-mortality associations across various demographic and clinical subgroups (Supplementary Figures S2 and S3). For all-cause mortality (Supplementary Figure S2), we consistently observed an inverse association between higher CALLY values and reduced mortality across sex, age, race/ethnicity, BMI, education, marital status, poverty income ratio, insurance, smoking, and comorbidities (all HRs < 1.00 for each unit increase in CALLY index, with most *P* < 0.05). Formal interaction testing indicated significant effect modification solely for alcohol use (*P* for interaction = 0.005), whereas all other interactions were non-significant (*P* > 0.05). Although point estimates appeared slightly stronger in males (HR 0.96, 95% CI: 0.94–0.98) compared with females (HR 0.97, 95% CI: 0.96–0.99), this difference was not statistically significant (*P* for interaction = 0.349) and should not be interpreted as true effect modification.

Regarding cardiovascular mortality (Supplementary Figure S3), we observed inverse associations across all subgroups (all HRs < 1.00 for each unit increase in CALLY index). No significant effect modification was detected for any covariate (all *P* for interaction > 0.05), including alcohol use (*P* = 0.928). Notably, although point estimates were quantitatively stronger in males (HR 0.91, 95% CI: 0.88–0.95) than in females (HR 0.96, 95% CI: 0.93–0.99), this observed difference did not reach statistical significance (*P* for interaction = 0.916). The inverse association of the CALLY index was consistently directionally aligned across various subgroups for both outcomes, thereby supporting its generalizability. Statistically significant effect modification was confined to alcohol use concerning all-cause mortality. For all other subgroup comparisons, the apparent differences, in the absence of significant interaction, likely reflect random variation or disparities in statistical power rather than genuine differential susceptibility.

To address potential reverse causality, we excluded participants who died within the first two years of follow-up and repeated the primary analyses (Supplementary Figure S4). The inverse association between CALLY and mortality remained robust. For all-cause mortality, HRs for Q4 versus Q1 were 0.318 (95% CI: 0.265–0.381) in the unadjusted Model 1, 0.534 (95% CI: 0.449–0.636) in Model 2, and 0.611 (95% CI: 0.505–0.738) in the fully adjusted Model 3 (P for trend < 0.001). For cardiovascular mortality, corresponding HRs were 0.241 (95% CI: 0.149–0.389), 0.408 (95% CI: 0.253–0.659), and 0.513 (95% CI: 0.326–0.808), respectively (P for trend < 0.001). These results indicate that reverse causation is unlikely to explain the observed inverse associations.

Temporal stability analysis revealed consistent results among participants interviewed from 1999 to 2002 (Supplementary Figure S5). In this subgroup, individuals in the highest quartile of CALLY maintained a significant association with reduced all-cause mortality (HRs ranging from 0.25 to 0.56 across models) and cardiovascular mortality (HRs ranging from 0.16 to 0.46 across models), showing significant dose-response trends (all *P* for trend < 0.001).

Adjustment for metabolic biomarkers was performed to assess the independence of the CALLY-mortality association from metabolic and oxidative stress pathways. Additional sensitivity analyses included adjustments for total bilirubin and serum uric acid (Supplementary Figure S6). Model 1 (fully adjusted plus total bilirubin) showed Q4 HRs of 0.57 (95% CI: 0.47–0.68) for all-cause mortality and 0.49 (95% CI: 0.38–0.64) for cardiovascular mortality. Model 2 (fully adjusted plus uric acid) indicated Q4 HRs of 0.59 (95% CI: 0.49–0.70) for all-cause mortality and 0.50 (95% CI: 0.39–0.64) for cardiovascular mortality. Model 3 (fully adjusted plus both biomarkers) demonstrated Q4 HRs of 0.58 (95% CI: 0.48–0.70) for all-cause mortality and 0.52 (95% CI: 0.40–0.67) for cardiovascular mortality. All associations remained statistically significant with consistent dose-response trends.

To evaluate potential collider bias, sensitivity analyses excluding disease variables yielded consistent results: Model 1 (excluding CVD and cancer): Q4 HR 0.58 (95% CI: 0.48–0.69) for all-cause and 0.49 (95% CI: 0.38–0.63) for cardiovascular mortality. Model 2 (additionally excluding hypertension and diabetes): Q4 HR 0.58 (95% CI: 0.49–0.69) and 0.49 (95% CI: 0.38–0.63), respectively (Supplementary Table S2). Collectively, these sensitivity analyses confirm that the inverse association between the CALLY index and mortality is robust, not influenced by reverse causation, stable across time periods, and independent of metabolic and oxidative stress markers.

## Discussion

This study extends prior observational research by implementing several methodological advances that, to our knowledge, have not been simultaneously applied in any previous NHANES-based CALLY study: (i) Fine-Gray competing risk models [21] to obtain valid cardiovascular mortality estimates in the presence of non-cardiovascular deaths; (ii) formal IDI [23] and NRI [24] quantification to assess whether the CALLY index improves risk prediction beyond 14 established risk factors; (iii) time-dependent ROC analysis with inverse probability of censoring weighting and optimism correction via 500 bootstrap resamples; (iv) restricted cubic splines [25] with cautious interpretation of extreme values; and (v) extensive sensitivity analyses addressing reverse causation, temporal stability, metabolic confounding, and collider bias. Although prior NHANES-based studies have examined the CALLY index in selected subpopulations (e.g., depression [9], prevalent CVD [10]), the present study is the first to apply this unified methodological framework in the general adult population. Although the Fine-Gray SHRs were similar in magnitude to those from standard Cox models, the competing risk approach provided more accurate estimates of the absolute cumulative incidence of cardiovascular death (e.g., 8.14% in Q1 vs. 3.56% in Q4 at 180 months), which cannot be obtained from Cox regression alone. This is particularly relevant in older populations where non-cardiovascular death is frequent.

The CALLY index exhibited a significant inverse association with all-cause and cardiovascular mortality, expanding on evidence beyond individual biomarkers such as CRP [26]. This aligns with the established prognostic importance of composite scores that capture the interplay among inflammation, nutrition, and immunity. Although previous studies validated the CALLY index in specific patient cohorts (chronic kidney disease [13], esophageal cancer [27], and acute cardiovascular conditions [15, 16]), data in the general population were limited. Recent NHANES-based studies by Zhang et al. (2026), Han et al. (2025), and Wu et al. (2025) examined the CALLY index in selected subpopulations but used conventional Cox regression without competing risk adjustment, did not quantify incremental predictive value using IDI/NRI, and lacked systematic time-dependent ROC analysis with optimism correction. The present study addressed these gaps by analyzing a large, well-defined cohort (*n* = 11,797) with the aforementioned methodological enhancements, thereby improving generalizability and rigor.

Unlike the PNI (restricted to nutritional and immune status) or CAR (reflecting only inflammation and hepatic synthetic function), the CALLY index concurrently captures all three interrelated axes. Although our analyses did not include direct head-to-head comparisons with all existing scores—partly because NHANES lacks some required variables at the same examination cycle—the independent association of the CALLY index after comprehensive adjustment suggests that its three-dimensional architecture may capture residual pathophysiological variance not fully explained by two-dimensional indices. Future studies should directly compare the CALLY index with the PNI, CAR, and SII within the same cohort to quantify any genuine incremental prognostic utility. Beyond theoretical construct differences, the CALLY index offers practical clinical utility as it is derived from three routinely available laboratory parameters (CRP, albumin, and lymphocyte count) at no additional cost or testing burden. In contrast, the PNI requires albumin and lymphocyte count but excludes inflammation; the CAR requires CRP and albumin but excludes immune status; and the SII requires platelet, neutrophil, and lymphocyte counts—parameters that, while routine, capture different pathophysiological dimensions. The value of the CALLY index lies not in replacing these scores but in serving as a readily accessible complementary marker that captures residual pathophysiological variance from three interconnected axes when integrated into multivariable risk models.

The predictive mechanism of the CALLY index may operate through an “inflammation-nutrition-immunity” axis. Elevated CRP reflects systemic inflammation and contributes to atherosclerosis progression [26, 28, 29]. Reduced serum albumin indicates insufficient nutritional reserves and altered vascular permeability, correlating with poor prognosis in chronic conditions [30, 31]. Lymphocyte depletion signifies immune dysfunction, which may compromise monitoring of plaque stability [32, 33]. The combined impact of these factors could create a pathophysiological synergy that worsens vascular injury [34–36]. These mechanisms are consistent with prior observations in disease-specific cohorts. However, these mechanistic interpretations remain speculative given the observational design and should be interpreted with caution.

Our RCS analyses identified distinct dose-response patterns. For all-cause mortality, the curve exhibited an approximately L-shaped pattern: risk declined steeply from low CALLY values and reached its lowest point in the moderate range (approximately CALLY 5–15, HR ≈ 0.8), after which it remained relatively flat across the bulk of the observed distribution. Beyond the 95th percentile (CALLY > 34.4), an apparent upturn was observed; however, the software-estimated nadir at CALLY = 23.6 was statistically unreliable, with the 95% confidence interval becoming extremely wide and crossing the null. This upturn was driven by fewer than 10 participants with CALLY > 50 and should be interpreted as a statistical artifact. For cardiovascular mortality, the curve declined monotonically within the observed data range (95% of participants had CALLY ≤ 36.9), with no evidence of a plateau. The software-estimated nadir at CALLY = 142.1 fell far outside the distribution of the data and is therefore clinically uninterpretable.

These results highlight the absence of a validated risk threshold for the CALLY index within the clinically relevant range. Rather than identifying a specific cut-point, our findings suggest a continuous inverse association in which moderate-to-high CALLY values are associated with optimal survival outcomes, with no clear upper boundary of benefit within the distribution of most participants. Any threshold derived from RCS should not be used for clinical decision-making.

**Residual Confounding and Interpretive Boundaries.** Despite comprehensive adjustment for demographic, socioeconomic, and clinical covariates, NHANES lacks data on several potentially important confounders. Information on physical activity, detailed dietary intake (beyond crude proxies), and medication use (e.g., statins, anti-inflammatory drugs) was not consistently available across survey cycles. These unmeasured factors may influence both CALLY components and mortality risk. For example, statin use reduces CRP and improves cardiovascular outcomes, whereas anti-inflammatory medications may alter lymphocyte counts. Physical inactivity and poor diet are associated with chronic inflammation and malnutrition. Importantly, unmeasured statin use would likely bias effect estimates toward the null (given its association with both lower CRP and improved outcomes), suggesting that our observed associations may be conservative. Although sensitivity analyses excluding disease variables and early deaths yielded consistent results, residual confounding cannot be fully excluded. Therefore, the observed associations should be interpreted as independent prognostic correlations, not causal effects.

Subgroup analyses indicated that the CALLY index maintained its predictive value across diverse demographic cohorts. For all-cause mortality, the association did not vary significantly by age (*P* for interaction = 0.866); HRs per unit increase ranged from 0.98 (95% CI: 0.96–1.00) in the 20–34 years group to 0.99 (95% CI: 0.98–1.00) in the > 64 years group. Similar consistency was observed for cardiovascular mortality (*P* for interaction = 0.397). These results align with previous NHANES studies by Han et al. (2025) [10] and Xie et al. (2025) [37], which used conventional Cox regression without competing risk adjustment or formal incremental utility assessment. The broad applicability of the CALLY index may stem from its capacity to capture both acute and chronic pathological processes.

An interaction between the CALLY index and alcohol consumption significantly influenced all-cause mortality (*P* for interaction = 0.005). The inverse association was more pronounced among current drinkers (HR = 0.96, 95% CI: 0.95–0.97) than non-drinkers (HR = 0.99, 95% CI: 0.98–1.00). However, alcohol was treated as a binary variable (yes/no), lacking information on quantity, frequency, or pattern. Thus, we could not explore dose-response relationships or differentiate moderate from heavy drinking. The observed interaction may be due to unmeasured confounders rather than a genuine biological effect [38]. This finding is purely hypothesis-generating and requires validation in studies with comprehensive alcohol exposure assessment.

The CALLY index showed significant associations with mortality across all BMI categories (underweight, normal weight, overweight, obese). Formal interaction testing indicated no statistically significant effect modification by BMI (*P* > 0.05). Although point estimates appeared slightly attenuated in the underweight category, confidence intervals substantially overlapped with other categories. Caution is advised against overinterpreting apparent BMI-specific differences without significant interaction. The obesity paradox—where traditional BMI inadequately predicts mortality in obese populations—does not appear to be addressed by the CALLY index, as effect sizes were comparable across BMI groups. Future research should investigate whether body composition measures (e.g., lean mass, visceral adiposity) alter the CALLY index’s predictive accuracy.

### Strengths and limitations

Key strengths include: (1) a large, nationally representative sample (*n* = 11,797) spanning six NHANES cycles (1999–2010) with rigorous survey weighting to ensure US population representativeness; (2) a three-tiered adjustment strategy progressively controlling for demographic, socioeconomic, and clinical confounders, guided by a DAG; (3) simultaneous application of competing risk models (Fine-Gray), formal incremental value quantification (IDI/NRI), and optimism-corrected time-dependent ROC analysis, providing a more rigorous evaluation than prior NHANES-based CALLY studies; and (4) comprehensive sensitivity analyses addressing reverse causation (excluding early deaths), temporal stability (1999–2002 subgroup), metabolic confounding (bilirubin and uric acid adjustment), and collider bias (disease variable exclusion).

Limitations include: (1) a single baseline CALLY measurement precluding assessment of temporal changes; (2) reliance on ICD-10 coding for cause of death, subject to misclassification; (3) residual confounding from unmeasured variables (physical activity, dietary intake, medication use), although sensitivity analyses excluding participants with baseline comorbidities yielded consistent results, and unmeasured statin use would likely bias toward the null; physical inactivity and poor diet are associated with chronic inflammation and malnutrition, whereas statin use reduces CRP and improves cardiovascular outcomes, suggesting that our observed associations may be conservative estimates; (4) binary alcohol classification precluding dose-response assessment; (5) RCS-derived risk thresholds that are statistically unstable and lack clinical validation; (6) modest standalone CALLY discrimination (AUC < 0.70), with high full-model AUC values primarily reflecting established risk factors rather than the CALLY index itself; and (7) a small IDI (< 0.5%) and non-significant NRI, indicating that the CALLY index does not meaningfully reclassify individual risk beyond established factors and should not be used as a standalone risk stratification tool.

## Conclusion

The CALLY index, integrating inflammatory, nutritional, and immune status, is independently associated with lower all-cause and cardiovascular mortality in US adults. This association is non-linear: for all-cause mortality, an L-shaped pattern within the observed range (risk decreasing from low to moderate values and plateauing thereafter, with an unstable upturn at extremes); for cardiovascular mortality, a monotonically decreasing non-linear relationship within the observed data range. Although exploratory analyses examined potential risk stratification thresholds, these estimates were statistically unstable and cannot be interpreted as validated cut-points. The stronger association observed among alcohol users generates hypotheses but should not influence behavioral recommendations owing to binary exposure assessment. Given the observational design and potential residual confounding, these findings should be interpreted as hypothesis-generating rather than definitive evidence for clinical implementation.

Future research should: (1) externally validate the incremental predictive value in separate cohorts; (2) prospectively validate risk stratification thresholds using repeated CALLY measurements; (3) conduct detailed alcohol exposure assessments to elucidate the observed interaction; (4) explore targeted intervention strategies; and (5) develop real-time detection technologies to facilitate clinical translation.

## Supplementary Information

Supplementary Material 1.

## Data Availability

Data Availability StatementThis study used publicly available data from the National Health and Nutrition Examination Survey (NHANES), conducted by the National Center for Health Statistics (NCHS). The datasets are freely accessible at https://www.cdc.gov/nchs/nhanes/index.htm. Mortality follow-up data were obtained through the NCHS Public-use Linked Mortality Files (https://www.cdc.gov/nchs/data-linkage/mortality-public). No new data were generated in this study. Analytical code is available from the corresponding authors upon reasonable request.
