# Supplementary material for: C-reactive protein-albumin-lymphocyte (CALLY) index and all-cause and cardiovascular mortality in the general population: a national health and nutrition examination survey analysis
Source: J Cardiothorac Surg. 2026 Jun 30;21:711. doi: 10.1186/s13019-026-04479-x (PMC13591912; doi:10.1186/s13019-026-04479-x)
Supplement: Supplementary file 1 [file 13019_2026_4479_MOESM1_ESM.docx]

Supporting information

**C-reactive Protein-Albumin-Lymphocyte Index Predicts All-Cause and Cardiovascular Mortality in the General Population: an analysis of data from the National Health and Nutrition Examination Survey**

**Table S1.** Collinearity Diagnostics for Covariates in the Multivariable Cox Regression Model

| **Variable** | **GVIF** | **Df** | **GVIF^(1/2Df)** |
| --- | --- | --- | --- |
| Age | 1.57 | 1 | 1.25 |
| Poverty income ratio | 1.32 | 1 | 1.15 |
| Race/ethnicity | 1.27 | 4 | 1.03 |
| Educational attainment | 1.26 | 1 | 1.12 |
| Insurance status | 1.26 | 1 | 1.12 |
| CALLY | 1.16 | 1 | 1.08 |
| Diabetes mellitus | 1.16 | 1 | 1.08 |
| Hypertension | 1.15 | 1 | 1.07 |
| CVD | 1.12 | 1 | 1.06 |
| Marital status | 1.12 | 1 | 1.06 |
| Smoking status | 1.11 | 1 | 1.05 |
| Cancer | 1.10 | 1 | 1.05 |
| Body mass index | 1.19 | 1 | 1.09 |
| Alcohol consumption | 1.07 | 1 | 1.03 |
| Sex | 1.04 | 1 | 1.02 |

Abbreviations: GVIF, generalized variance inflation factor; Df, degrees of freedom; CALLY, C-reactive Protein-Albumin-Lymphocyte Index; CVD, cardiovascular disease.

Note: All GVIF values < 5.0 and GVIF^(1/2Df) values < 2.0 indicate no substantial multicollinearity among covariates.

**Table S2.** Sensitivity analyses excluding disease variables to address overadjustment and collider bias.

| **Subgroup** | **Model 1** | | **Model 2** | |
| --- | --- | --- | --- | --- |
|  | **HR (95% CI)** | **P** | **HR (95% CI)** | **P** |
| **All-cause mortality** | | | | |
| Q1 | Reference | | Reference | |
| Q2 | 0.722 (0.626,0.833) | **<0.001** | 0.721 (0.625,0.831) | **<0.001** |
| Q3 | 0.674 (0.574,0.793) | **<0.001** | 0.681 (0.580,0.799) | **<0.001** |
| Q4 | 0.577 (0.484,0.687) | **<0.001** | 0.582 (0.490,0.691) | **<0.001** |
| **Cardiovascular mortality** | | | | |
| Q1 | Reference | | Reference | |
| Q2 | 0.654 (0.489,0.874) | 0.004 | 0.659 (0.493,0.881) | 0.005 |
| Q3 | 0.590 (0.448,0.776) | **<0.001** | 0.595 (0.456,0.777) | **<0.001** |
| Q4 | 0.488 (0.379,0.628) | **<0.001** | 0.488 (0.380,0.627) | **<0.001** |

To evaluate whether adjustment for prevalent diseases introduced overadjustment or collider bias, Cox proportional hazards regression models were fitted excluding disease variables that may act as mediators or colliders in the causal pathway between CALLY index and mortality. Model 1 adjusted for all covariates in the primary fully-adjusted model (age, sex, race/ethnicity, BMI, education, PIR, marital status, smoking, alcohol use, insurance status) but excluded cardiovascular disease (CVD) and cancer. Model 2 additionally excluded hypertension and diabetes, representing a conservative approach that removes all major metabolic and disease conditions.

**Table S3.**

| **Cumulative Incidence Function (CIF) by CALLY Quartile** | | | | | | | | | | |
| --- | --- | --- | --- | --- | --- | --- | --- | --- | --- | --- |
|  | | | | | | | | | | |
| **Time** | **Cardiovascular death** | | | | **Gray's test** | **Competing event (Other-cause death)** | | | | **Gray's test** |
| **(months)** | **Q1** | **Q2** | **Q3** | **Q4** | ***P*** | **Q1** | **Q2** | **Q3** | **Q4** | ***P*** |
| **0** | 0.00% | 0.00% | 0.00% | 0.00% | **< 0.001** | 0.00% | 0.00% | 0.00% | 0.00% | **< 0.001** |
| **10** | 0.47% | 0.07% | 0.03% | 0.03% |  | 1.15% | 0.41% | 0.30% | 0.14% |  |
| **20** | 0.81% | 0.24% | 0.30% | 0.14% |  | 2.41% | 1.19% | 0.88% | 0.61% |  |
| **30** | 1.15% | 0.58% | 0.61% | 0.31% |  | 4.30% | 2.14% | 1.79% | 1.09% |  |
| **40** | 1.73% | 0.81% | 0.84% | 0.41% |  | 5.66% | 2.95% | 2.57% | 1.57% |  |
| **50** | 2.34% | 1.32% | 1.01% | 0.61% |  | 6.78% | 4.00% | 3.11% | 2.04% |  |
| **60** | 2.64% | 1.80% | 1.38% | 0.72% |  | 7.76% | 4.75% | 3.88% | 2.62% |  |
| **70** | 3.08% | 2.07% | 1.65% | 0.78% |  | 9.25% | 5.90% | 5.03% | 3.20% |  |
| **80** | 3.52% | 2.44% | 1.93% | 0.92% |  | 10.47% | 7.23% | 5.98% | 4.05% |  |
| **90** | 4.13% | 2.65% | 2.36% | 1.06% |  | 11.79% | 8.41% | 6.79% | 4.80% |  |
| **100** | 4.54% | 2.88% | 2.67% | 1.26% |  | 12.78% | 9.57% | 7.57% | 5.41% |  |
| **110** | 5.02% | 3.19% | 2.97% | 1.50% |  | 14.37% | 10.55% | 8.51% | 6.16% |  |
| **120** | 5.51% | 3.70% | 3.15% | 1.57% |  | 15.32% | 11.57% | 9.44% | 6.78% |  |
| **130** | 6.05% | 4.13% | 3.31% | 1.85% |  | 16.23% | 12.65% | 10.73% | 7.61% |  |
| **140** | 6.32% | 4.62% | 3.55% | 2.04% |  | 17.41% | 13.70% | 11.37% | 8.14% |  |
| **150** | 6.77% | 5.19% | 3.99% | 2.26% |  | 18.44% | 14.82% | 12.43% | 8.69% |  |
| **160** | 7.20% | 5.54% | 4.30% | 2.70% |  | 19.12% | 15.99% | 13.38% | 9.14% |  |
| **170** | 7.59% | 5.87% | 4.38% | 2.93% |  | 20.66% | 16.92% | 14.48% | 9.91% |  |
| **180** | 8.14% | 6.19% | 4.91% | 3.56% |  | 21.78% | 18.57% | 15.36% | 10.18% |  |
| CIF estimated by Aalen-Johansen method. Group comparison by Gray's test. Competing event: death from causes other than CVD. | | | | | | | | | | |
| Data: NHANES 1999-2010 | | |  |  |  |  |  |  |  |  |

**Figure S1**. The distribution of the continuous variables in this study. BMI: body mass index; CALLY: C-reactive Protein-Albumin-Lymphocyte Index.

**Figure S2**. Association between CALLY and all-cause mortality among adults in different subgroups. CALLY: C-reactive Protein-Albumin-Lymphocyte Index; BMI: body mass index; PIR: poverty income ratio; CVD: cardiovascular disease; DM: diabetes mellitus; HBP: hypertension. Formal interaction testing indicated significant effect modification for alcohol use (P = 0.005); all other interactions were non-significant (P > 0.05).

**Figure S3.** Association between CALLY and Cardiovascular mortality among adults in different subgroups. CALLY: C-reactive Protein-Albumin-Lymphocyte Index; BMI: body mass index; PIR: poverty income ratio; DM: diabetes mellitus; HBP: hypertension. Formal interaction testing indicated no significant effect modification for any covariate (all P for interaction > 0.05), including alcohol use (P = 0.928).

**Figure S4**. Sensitivity analyses to evaluate the association between CALLY and the mortality outcomes of adults by excluding participants who died within 2 years.

Abbreviation: HR: hazard ratio; CI: confidence interval; Q: quartile. CALLY: C-reactive Protein-Albumin-Lymphocyte Index; BMI: body mass index; PIR: poverty income ratio; CVD: cardiovascular disease; DM: diabetes mellitus.

Model 1: unadjusted; Model 2: adjusted for age, gender, race; Model 3: adjusted for age, gender, race, marital status, educational attainment, BMI, PIR, smoking status, alcohol use, CVD, hypertension, cancer, diabetes, and, Insurance status.

**Figure S5**. Sensitivity analyses to evaluate the association between CALLY and the mortality outcomes of adults by only including participants from 1999 to 2002.

Abbreviation: HR: hazard ratio; CI: confidence interval; Q: quartile. CALLY: C-reactive Protein-Albumin-Lymphocyte Index; BMI: body mass index; PIR: poverty income ratio; CVD: cardiovascular disease; DM: diabetes mellitus; HBP: hypertension.

Model 1: unadjusted; Model 2: adjusted for age, gender, race; Model 3: adjusted for age, gender, race, marital status, educational attainment, BMI, PIR, smoking status, alcohol use, CVD, HBP, cancer, diabetes, and, Insurance status.

**Figure S6**. Sensitivity analyses to evaluate the association between CALLY and the mortality outcomes of adults with additional adjustment for total bilirubin and uric acid.

Abbreviation: HR: hazard ratio; CI: confidence interval; Q: quartile. CALLY: C-reactive Protein-Albumin-Lymphocyte Index; BMI: body mass index; PIR: poverty income ratio; CVD: cardiovascular disease; DM: diabetes mellitus.

Model 1: adjusted for age, gender, race, marital status, educational attainment, BMI, PIR, smoking status, alcohol use, CVD, hypertension, cancer, diabetes, Insurance status, and total bilirubin; Model 2: adjusted for age, gender, race, marital status, educational attainment, BMI, PIR, smoking status, alcohol use, CVD, hypertension, cancer, diabetes, insurance status, and uric acid; Model 3: fully adjusted model plus both total bilirubin and uric acid.

**Figure S7**.

Fig S7**.** Time-dependent receiver operating characteristic (ROC) curves for the multivariable model based on 70% random subsampling of the cohort for internal validation. (A) All-cause mortality; (B) Cardiovascular mortality. The multivariable model was adjusted for age, sex, race/ethnicity, body mass index, educational attainment, poverty income ratio, marital status, smoking status, alcohol consumption, hypertension, diabetes mellitus, cardiovascular disease, cancer, and insurance coverage. Optimism-corrected area under the curve (AUC) values were derived from 500 bootstrap resamples. AUC values are reported for three time points (36, 60, and 120 months).

**Figure S8**.  Directed acyclic graph (DAG) for covariate selection.

*Exposure*: CALLY index. *Outcome*: all‑cause mortality.
*Adjusted variables* (confounders, each with arrows pointing to both CALLY and mortality): age, sex, BMI, smoking, alcohol, baseline CVD, cancer, diabetes, hypertension, race, education, insurance, marital status.
The DAG implies that adjustment for these confounders is sufficient to estimate the total effect of CALLY on mortality.

**Figure S9.**

Figure S9. Incremental predictive value of the CALLY index beyond established risk factors. Integrated Discrimination Improvement (IDI), Net Reclassification Improvement (NRI), and median improvement in predicted probabilities are shown for all-cause mortality and cardiovascular mortality. Error bars represent 95% confidence intervals derived from 500 bootstrap resamples. IDI was statistically significant for both outcomes (P = 0.007), while NRI did not reach statistical significance (P = 0.272 for all-cause; P = 0.399 for cardiovascular). Median improvement was significant for both outcomes (P < 0.001).
